# New Books

**Published:** 2006-01

**Agricultural Biodiversity and Biotechnology in Economic Development**

Joseph Cooper, Leslie Marie Lipper, David Zilberman

New York:Springer, 2005. 480 pp. ISBN: 0-387-25407-2, $99

**Air Quality in Airplane Cabins and Similar Enclosed Spaces, Vol. 4: Air Pollution, Part H**

Martin B. Hocking, Diana Hocking, eds.

New York:Springer, 2005. 410 pp. ISBN: 3-540-25019-0, $179

**Algorithms in Bioinformatics**

Rita Casadio, Gene Myers, eds.

New York:Springer, 2005. 436 pp. ISBN: 3-540-29008-7, $78

**Appraising Sustainable Development: Water Management and Environmental Challenges**

Asit K. Biswas, Cecilia Tortajada, eds.

New York:Oxford University Press, 2005. 242 pp. ISBN: 0-19-566891-X, $34.50

**Bioethics: An Introduction for the Biosciences**

Ben Mepham

New York:Oxford University Press, 2005. 408 pp. ISBN: 0-19-926715-4, $39.95

**Bioinformatics and Computational Biology Solutions Using R and Bioconductor**

R. Gentleman, V. Carey, W. Huber, R. Irizarry, S. Dudoit, eds.

New York:Springer, 2005. 452 pp. ISBN: 0-387-25146-4, $89.95

**Data Mining and Knowledge Discovery Handbook**

Oded Maimon, Lior Rokach

New York:Springer, 2005. 1,383 pp. ISBN: 0-387-24435-2, $199

**DNA Methylation, Epigenetics and Metastasis**

Manel Esteller, ed.

New York:Springer, 2005. 310 pp. ISBN: 1-4020-3641-8, $149

**Environmental Issues in Latin America and the Caribbean**

Aldemaro Romero, Sarah E. West, eds.

New York:Springer, 2005. 299 pp. ISBN: 1-4020-3773-2, $129

**Genes on the Menu: Facts for Knowledge-Based Decisions**

Paul Pechan, Gert de Vries

New York:Springer, 2005. 217 pp. ISBN: 3-540-20178-5, $39.95

**Globalization and Urban Development**

Harry W. Richardson, Chang-Hee C. Bae, eds.

New York:Springer, 2005. 321 pp. ISBN: 3-540-22362-2, $99

**Greenhouse Warming and Nuclear Hazards: A Series of Essays and Research Papers**

Peter Fong

Hackensack, NJ:World Scientific Publishing, 2005. 276 pp. ISBN: 981-256-422-5, $48

**Oceans and Health: Pathogens in the Marine Environment**

Shimshon S. Belkin, Rita R. Colwell, eds.

New York:Springer, 2005. 464 pp. ISBN: 0-387-23708-9, $89.95

**Proteomics in Cancer Research**

Daniel C. Liebler, ed.

Hoboken, NJ:John Wiley & Sons, 2005. 201 pp. ISBN: 0-471-44476-6, $135

**Small-scale Freshwater Toxicity Investigations: Vol. 1—Toxicity Test Methods**

Christian Blaise, Jean-François Férard, eds.

New York:Springer, 2005. 551 pp. ISBN: 1-4020-3119-X, $129

**Sustainability and Cities: Concept and Assessment**

Ooi Giok Ling

Hackensack, NJ:World Scientific Publishing, 2005. 252 pp. ISBN: 981-256-163-3, $48

**The Obesity Epidemic: Science, Morality and Ideaology**

Michael Gard, Jan Wright

New York:Routledge, 2005. 218 pp. ISBN: 0-415-31895-5, $125

**The Toxicology of Fishes**

Richard T. Di Giulio, David E. Hinton, eds.

New York:Routledge, 2005. 752 pp. ISBN: 0-415-24868-X, $138.48

**Toxicology of Organophosphate and Carbamate Compounds**

Ramesh C. Gupta, ed.

Burlington, MA:Elsevier, 2005. 768 pp. ISBN: 0-12-088523-9, $139.95

